# Combatting type 2 diabetes by turning up the heat

**DOI:** 10.1007/s00125-016-4068-3

**Published:** 2016-09-03

**Authors:** Patrick Schrauwen, Wouter D. van Marken Lichtenbelt

**Affiliations:** Department of Human Biology and Human Movement Sciences, NUTRIM School for Nutrition and Translational Research in Metabolism, Maastricht University Medical Center, P.O. BOX 616, 6200MD Maastricht, the Netherlands

**Keywords:** Brown adipose tissue, Cold-induced thermogenesis, Diabetes, Energy turnover, Review

## Abstract

In our westernised society, the level of physical activity is low. Interventions that increase energy expenditure are generally associated with an improvement in metabolic health. Exercise and exercise training increase energy metabolism and are considered to be among the best strategies for prevention of type 2 diabetes mellitus. More recently, cold exposure has been suggested to have a therapeutic value in type 2 diabetes. At a cellular level, there is evidence that increasing the turnover of cellular substrates such as fatty acids is associated with preventive effects against lipid-induced insulin resistance. Cellular energy sensors may underlie the effects linking energy turnover with metabolic health effects. Here we review data supporting the hypothesis that increasing energy and substrate turnover has beneficial effects on insulin sensitivity and should be considered a target for the prevention and treatment of type 2 diabetes.

**Electronic supplementary material:**

The online version of this article (doi:10.1007/s00125-016-4068-3) contains a slideset of the figures for download, which is available to authorised users.

## Introduction

Obesity is the major risk factor for the development of type 2 diabetes mellitus. It is not only characterised by an increased storage of fat in subcutaneous white adipose tissue but is also associated with increased storage of fat in non-adipose tissues, such as muscle and liver. This so-called ectopic fat accumulation is thought to lead to the development of insulin resistance, an early hallmark in the development of diabetes [1, 2]. Therefore, weight loss is the first-choice preventive and therapeutic intervention for type 2 diabetes; indeed, a reduction in body weight leads to improvement in insulin sensitivity and weight reduction is a good strategy for preventing the development of diabetes. However, long-term maintenance of a healthy weight is difficult.

Although whole-body energy balance is determined by both energy intake and energy expenditure, the window of opportunity for achieving a negative energy balance (and thus weight loss) is much larger when energy intake is challenged. A diet very low in energy reduces energy intake by up to 70–80%, whereas a similar percentage increase in energy expenditure would require a very substantial amount of physical activity, such as that achieved by participating in competitive sports. Indeed, exercise training programmes do not lead to major weight loss; for example, it has been shown that jogging the equivalent of 20 miles per week or walking 12 miles per week only resulted in a weight loss of 3.5 kg and 1.1 kg, respectively, after an 8 month training period [3]. Consequently, over recent decades most of the research in the obesity field has focused on reducing energy intake to combat obesity and type 2 diabetes, and the role of energy expenditure in metabolic health has been underappreciated and understudied. However, it is becoming increasingly evident that enhancing energy metabolism per se can counterbalance the metabolic consequences of obesity and that increasing energy turnover is an important target in the prevention of obesity-related metabolic disturbances, such as insulin resistance. For example, even though exercise has only minor effect on body weight, it is known to have a beneficial effect on many metabolism-related diseases such as type 2 diabetes [4], cardiovascular disease [5], the metabolic syndrome [6] and even cancer [7]. This notion is underscored further by the recent evidence that breaking sedentary time can have major effects on insulin sensitivity and metabolic profile [8–12], suggesting that even small increases in physical activity levels can have a major impact on health. Similarly, other environmental factors such as cold exposure can have substantial boosting effects on energy metabolism and are associated with metabolic health effects (see below). Here we review the evidence, focusing on human intervention studies, suggesting that increasing energy turnover by exercise and/or cold exposure can offset obesity-related insulin resistance and be a preventive strategy for type 2 diabetes mellitus.

## Defining components of human energy metabolism

### Heat production

In the (human) body, almost all energy is ultimately converted into heat [13], and heat production is the gold standard for the measurement of energy expenditure under resting conditions. Apart from the performance of external work, energy in the body is used in the form of ATP for all metabolic processes that have heat production as a final end-product. The resting metabolic rate (RMR) is largely determined by the sum and the efficiency of these processes; for example, the continuous cycle of protein synthesis and breakdown is an energy-requiring process, and an increase in protein turnover results in the extra production of heat and thus elevated energy expenditure. The basal metabolic rate (BMR) is defined as the fasting resting energy expenditure in the morning in thermoneutral conditions; under less strict conditions this is the RMR [14]. The metabolic rate during sleep is slightly lower than the BMR, as being awake requires energy. As well as the BMR there is diet-induced thermogenesis (DIT), also known as thermogenic effect of food, which is the amount of extra heat related to the digestion, absorption and intermediary processing of food. DIT is in the order of magnitude of 5–10% of the total energy intake under energy balance conditions. Physical activity (exercise) also increases heat production, due to the increased energy demand necessary to perform the external work, particularly since only 20–25% of the produced energy can be used for external work (i.e. low mechanical energy efficiency with 75–80% of produced energy lost as heat) [15]. Although physical activity energy expenditure can be large—up to 4–5 times the BMR—it is highly variable both within and between individuals. Only low levels of physical activity can be sustained for long periods and in the general population intensive exercise only tends to consist of short bouts. As a result, physical activity energy expenditure typically forms about 30% of the 24 h energy expenditure in free-living individuals [16].

### Heat regulation

Heat produced by the above processes is lost via respiration (evaporation) and via the skin (conduction, convection, radiation and evaporation). Under resting, thermoneutral conditions there is heat balance at a core body temperature of around 37°C, so no extra heat production is needed. However, if the environmental temperature is above or below the thermoneutral zone heat production increases [17]. This increased production of heat can be achieved by increased turnover of metabolic processes and induction of futile cycles, by muscle contraction (shivering) and also by so-called mitochondrial uncoupling. Thus in the cell and mitochondria, energy substrates are broken down in processes such as β-oxidation, glycolysis and the tricarboxylic acid cycle, ultimately leading to the build-up of a proton gradient over the inner mitochondrial membrane and ATP generation [18]. Mitochondrial uncoupling lowers the proton gradient without ATP formation, thereby reducing energy efficiency and indirectly stimulating heat production. Shivering can increase energy expenditure to up to four times the BMR [19], but cannot be maintained for prolonged periods as it is uncomfortable, decreases coordination and results in muscle fatigue. Non-shivering thermogenesis (NST), on the other hand, among other processes occurring via regulated mitochondrial uncoupling, can be sustained, is not uncomfortable (it is insensible) and does not affect coordination. The maximal reported NST is 40% of the RMR [20] but varies between individuals; in healthy lean individuals it ranges from 0% to 30% of the RMR [21–23].

Unless physical activity levels are raised to those seen in competitive sports, whole-body 24 h energy expenditure can typically only be sustainably elevated by ∼10–20% in humans. Nevertheless, many of the interventions that increase energy expenditure have marked metabolic health effects. Given the relatively minor effects on whole-body 24 h energy expenditure, which is often also compensated for by increased energy intake, the beneficial effects of interventions such as exercise and cold exposure cannot be attributed to weight loss. It is worth noting that this does not imply that energy expenditure has no role in body-weight regulation since a small increase or decrease in the BMR of 5% could theoretically lead to a reduction in body weight of ∼5–10 kg in a year, if not compensated by other means [24]. Indeed, a low RMR has been shown to be a risk factor for the development of obesity [25]. Nevertheless, most intervention studies in which energy expenditure is elevated do show beneficial metabolic health effects without changes in body weight.

## Evidence that enhancing energy turnover improves insulin sensitivity: mitochondrial uncoupling

**Fig. 1:**
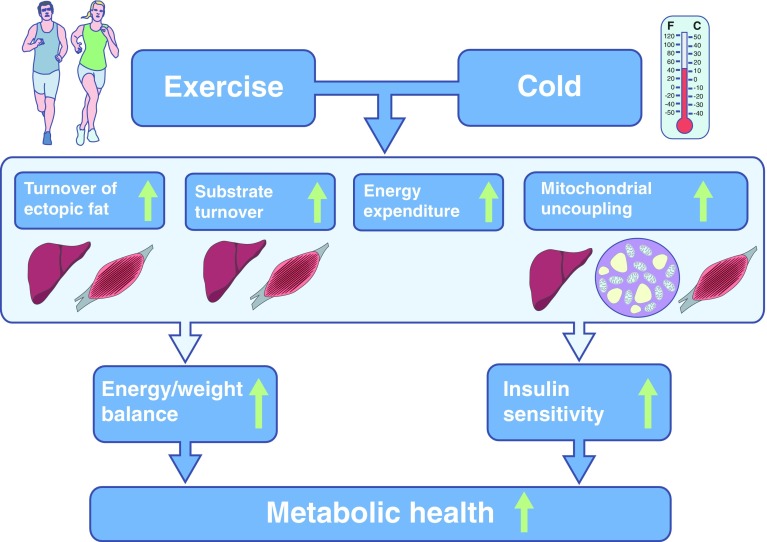
Schematic overview of how interventions that target energy turnover, such as exercise and cold exposure, may affect metabolic health. Although the exact mechanisms are unknown, cold exposure and exercise have been shown to increase or improve the following factors (indicated by green arrows): turnover of fat in ectopic sites (liver, skeletal muscle); mitochondrial function and mitochondrial uncoupling (in liver, BAT and skeletal muscle); energy expenditure (in all tissues); and fatty acid turnover (in liver and skeletal muscle). These effects may lead to alterations in energy balance and body weight but may also lead to improvement in insulin sensitivity independent of change in body weight

If enhancing energy turnover does not lead to major weight loss, the question arises as to what mechanism can explain the beneficial health effects of enhanced energy turnover (Fig. 1). A variety of mechanisms, such as muscle remodelling, sympathetic nervous system activation, hormonal changes and mitochondrial biogenesis, have been suggested to underlie the metabolic health effects of specific interventions like exercise and exposure to cold, and it is difficult to prove that these effects are direct. Nevertheless, there is clear evidence that boosting energy turnover may have a direct beneficial health effect as it is underscored by studies in which energy turnover is increased by inducing mitochondrial uncoupling. Overexpression of the mitochondrial uncoupling proteins UCP1 or UCP3 in skeletal muscle increases energy expenditure and improves insulin sensitivity [26–28]. Mitochondrial uncoupling can, apart from exercise training or cold exposure, also be increased by chemical agents such as 2,4-dinitrophenol (DNP), although the use of DNP in humans was banned in the 1930s after several cases of lethal hyperthermia. Recently, however, it was shown that targeting DNP towards the liver reduced hypertriacylglycerolaemia, fatty liver and whole-body insulin resistance in high-fat-fed rats and decreased hyperglycaemia in a rat model of type 2 diabetes [29]. Similarly, it was recently shown that niclosamide ethanolamine salt (NEN) induced mitochondrial uncoupling in mice, increased energy expenditure and lipid metabolism and was efficacious in preventing and treating hepatic steatosis and insulin resistance induced by a high-fat diet [30]. Moreover, NEN improved glycaemic control and delayed disease progression in *db*/*db* mice [30]. So far, drugs that induce mitochondrial uncoupling specifically in certain tissues are not available for use in humans. Nevertheless, it has been shown that insulin-sensitive endurance-trained athletes display elevated mitochondrial uncoupling and enhanced substrate turnover [31]. Recently, we showed that endurance-trained athletes have an increased sensitivity to fatty acid-induced uncoupling, together with elevated levels of the mitochondrial protein adenine nucleotide translocase 1 (ANT1), which is thought to be involved in facilitating fatty acid-induced uncoupling [32, 33]. Interestingly, the level of fatty acid-induced uncoupling was related to the level of insulin sensitivity and reducing ANT1 levels in C2C12 muscle cells reduced fatty acid-induced uncoupling and insulin-stimulated glucose uptake [34]. In addition, thyroid hormone has been shown to induce mitochondrial uncoupling in skeletal muscle and to increase thermogenesis and RMR in humans [35]. Together, these results suggest that mitochondrial uncoupling, likely to lead to enhanced energy turnover, improves glucose homeostasis in rodents and humans.

## Link between energy turnover, ectopic fat and insulin sensitivity

### Athlete’s paradox

As outlined above, one of the major determinants of obesity-associated metabolic complications is the location of the storage of excess fat, with fat accumulation in metabolically active tissues such as muscle and liver leading to insulin resistance of these tissues [1, 2]. One could thus speculate that enhancing mitochondrial uncoupling, thereby increasing cellular energy expenditure, would lead to the burning-off of intracellular fat and reduction of ectopic fat stores. Although this may be true for the studies mentioned above, improvement in metabolic health via energy metabolism-enhancing interventions do not necessarily require a reduction in ectopic fat. The best known example of this is the so-called athletes paradox—even though accumulation of fat in skeletal muscle is associated with insulin resistance, highly insulin-sensitive endurance-trained athletes also have very high levels of intramyocellular lipid [36]. This discrepancy has often been explained by the notion that it is fatty acid intermediates (such as ceramides and diacylglycerol) and not intramyocellular triacylglycerol (the major form in which fat is accumulated in lipid droplets) that lead to insulin resistance, and that these intermediates accumulate when the mitochondrial oxidative capacity of the muscle is low [37, 38]. However, endurance-trained athletes also have elevated levels of diacylglycerol [39], and muscle-specific overexpression of diacylglycerol acyltransferase (the enzyme converting diacylglycerol into triacylglycerol) leads to improved insulin sensitivity despite elevated diacylglycerol levels [40]. Furthermore, the concept that simply having more mitochondria could counterbalance lipid-induced skeletal muscle insulin resistance is not entirely correct, as it has been shown that overexpression of peroxisome proliferator-activated receptor γ coactivator 1 (PGC1) in mice leads to an improved mitochondrial function, yet such mice are not protected from high-fat-diet-induced insulin resistance [41]. However, when these mice are stimulated to become physically active, and thereby use their capacity for elevated energy and substrate turnover, they are protected from insulin resistance [42]. Interestingly in that context, PGC1 not only regulates mitochondrial function but is also involved in the transcriptional regulation of lipid droplet coating proteins, which contribute to the regulation of fatty acid delivery to the mitochondria [43]. This suggests that intracellular transcriptional programs exist that not only regulate cellular oxidative capacity but are also tightly involved in the regulation of substrate release and delivery, thereby laying the basis for efficient energy and substrate turnover. In accordance, overexpression of the lipid droplet coating proteins perilipin (PLIN) 2 or PLIN5, which are involved in the fine-tuning of fatty acid release for mitochondrial use, in skeletal muscle or liver also prevents lipid-induced insulin resistance, despite increased fat accumulation [44–46].

### Energy turnover

Human studies also show that it is not the level of lipid or intermediates per se that leads to insulin resistance, but that it is the turnover of these substrates that determines whether fat accumulation leads to insulin resistance (Fig. 1). Thus, we have previously shown that in individuals with type 2 diabetes the capacity to convert fatty acids into inert triacylglycerols is reduced when compared with obese controls, suggesting that triacylglycerol turnover is reduced. In accordance, Listenberger et al [47] showed that the capacity for triacylglycerol accumulation is an important determinant of fatty-acid-induced insulin resistance in skeletal muscle cells and that impairing triacylglycerol synthesis induced lipotoxicity. These in vitro and ex vivo findings are in accordance with those of Perreault et al [48] who elegantly determined fractional synthesis rate and intramyocellular lipid concentration in obese volunteers with impaired glucose tolerance vs BMI-matched normoglycaemic controls. Interestingly, it was found that intramyocellular lipid concentration was higher but fractional synthesis rate was lower in the glucose-intolerant individuals. These findings were confirmed in a later report by the same authors, although the finding could only be verified in men and not in women [49]. Also in liver, hepatic fat is not detrimental per se as it is known that fat in the liver has the important physiological function of temporarily buffering circulating fatty acids and triacylglycerols. However, chronic oversupply of fat to the liver, such as in obesity, leads to hepatic steatosis, increased VLDL-triacylglycerol production, hepatic insulin resistance and ultimately hepatic failure [50, 51]. Taken together, these findings suggest that the link between cellular fat accumulation and insulin sensitivity is not straightforward, but depends on the tight balance between cellular fat storage capacity and mitochondrial oxidative capacity, and specifically the fine-tuned regulation of the turnover of fatty acids. It may be hypothesised that increasing energy turnover—by exercise, cold exposure, thyroid hormone, uncoupling or other interventions—may improve insulin sensitivity by enhancing the turnover of fat in ectopic fat stores, and thereby preventing deleterious effects of excessive ectopic fat storage (Fig. 1). However, this concept would need to be tested.

## Cellular mechanisms linking energy turnover to metabolic adaptations

**Fig. 2:**
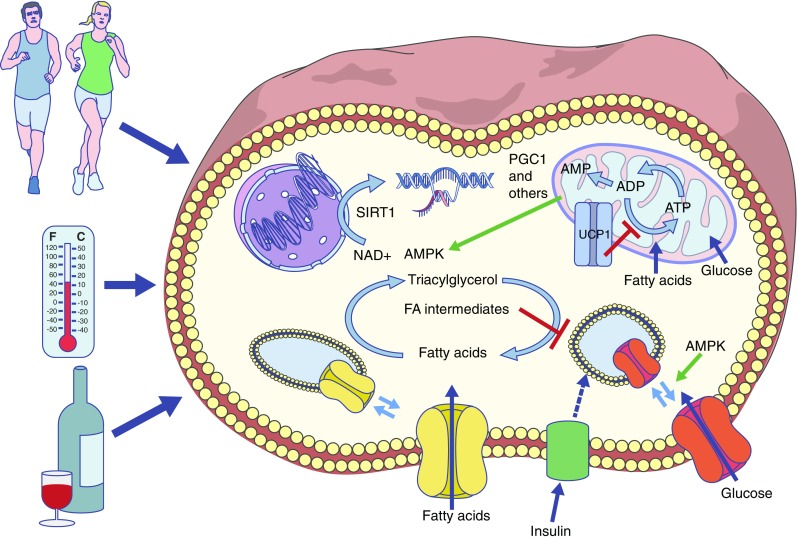
Schematic representation of cellular mechanisms linking energy-boosting interventions and metabolic health effects. Fatty acids and glucose are taken up into muscle via their respective transporters (CD36 [yellow transporter] and GLUT4 [red transporter]), which translocate from intracellular stores upon AMPK activation. Fatty acids and glucose can be used as substrates inside mitochondria for ATP generation, or be stored as glycogen and triacylglycerol. In the process of lipid storage, lipotoxic fatty acid intermediates can be formed that inhibit GLUT4 translocation. Mitochondrial uncoupling, via uncoupling proteins (UCP1), affects the efficiency by which ATP is formed. When ATP demand exceeds ATP formation, ADP levels increase and AMP may be formed, leading to activation of AMPK. Exercise, cold exposure and dietary resveratrol (found in red wine, among other things) may affect molecular pathways that activate AMPK and SIRT1, which in turn leads to the activation of PGC1, a transcription factor involved in the regulation of mitochondrial metabolism. As a result, mitochondrial uncoupling or enhanced fatty acid turnover may occur, thereby preventing the negative effects of cellular substrate overload

At the cellular level, turnover of energy and substrates is driven by energy demand, either because ATP is needed for cellular processes or because the efficiency of ATP formation is reduced by mitochondrial uncoupling (Fig. 2). In the cell, an increase in energy use can lead to alterations in the AMP/ATP and NAD^+^/NADH ratios resulting in the activation of among others AMPK (Fig. 2) [52–54] and sirtuin 1 (SIRT1) [55]. These energy sensors are strongly involved in the regulation of cellular energy metabolism, and activation of these factors is associated with metabolic health effects, as has been reviewed extensively elsewhere [54, 56–58]. Thus, these energy sensors provide a molecular explanation for how boosting energy turnover could be beneficial for the prevention and treatment of type 2 diabetes mellitus. They are also direct targets for pharmacological and nutritional approaches for treating/preventing diabetes. In fact, the most widely prescribed glucose-lowering drug, metformin, has been suggested to work via the induction of energy stress in the cell and thereby activation of AMPK [59], although the exact working mechanism is still the subject of debate [60, 61].

## Human interventions that increase energy turnover

### Exercise

Adding exercise to daily activities enhances whole-body energy expenditure and during exercise energy expenditure can be elevated several fold. It is also known that exercise training is one of the best strategies for the prevention or treatment of type 2 diabetes.

#### Acute exercise

Acute exercise increases glucose homeostasis through the activation of AMPK—the energy sensor of the cell—leading to the translocation of GLUT4 to the cell membrane, an alternative to insulin-induced GLUT4 translocation (Fig. 2) [62]. Furthermore, and in line with the previous outlined hypothesis, it was demonstrated by Schenk and Horowitz [63] that one bout of acute exercise could reverse lipid-induced insulin resistance in humans, and that this was accompanied by an increased partitioning of excess fatty acids towards triacylglycerol synthesis in muscle. Therefore, by increasing energy turnover and AMPK activation inside muscle, acute exercise can acutely and beneficially affect ‘insulin sensitivity’, at least in skeletal muscle.

#### Chronic exercise

Chronic exercise most likely leads to skeletal muscle adaptation via chronic activation of AMPK, although many other mechanisms have been suggested to explain why exercise-trained skeletal muscle has an improved insulin sensitivity. We will not review these mechanisms here, as they have been reviewed extensively elsewhere [64–67]. However, one of the best-described adaptations to chronic exercise training is an improvement in muscle oxidative capacity and mitochondrial function. Indeed, we [68, 69] and others [70–73] have shown that in type 2 diabetes patients an exercise-induced improvement in insulin sensitivity is associated with an improvement in mitochondrial function. In line with the hypothesis that elevated substrate turnover associates with insulin sensitivity, exercise training for weeks to months results in an improvement in insulin sensitivity not only in healthy individuals but also in individuals who are obese and have type 2 diabetes, even though levels of intramyocellular lipid are not lowered. In fact, intramyocellular lipid is often further increased [43]. Interestingly, endurance-trained athletes have higher levels of PLINs [43], suggesting improved regulation of substrate turnover with exercise training. Indeed, Bergman et al [74] showed that the fractional synthesis rate of intramyocellular triacyglycerol was significantly increased in endurance-trained male cyclists when compared with age- and BMI-matched sedentary men.

More recently, attention has also shifted towards determining whether, and if so how, exercise can also be beneficial for other metabolic tissues. For example, it has been shown that exercise training can have beneficial effects on hepatic metabolism [75–77]. This may partly explain the beneficial effects of training on circulating triacylglycerol and cholesterol levels and on postprandial lipid metabolism, important factors in the development of obesity-related metabolic complications [78]. Interestingly, also in the liver a reduced energy metabolism has been linked to hepatic steatosis and hepatic insulin resistance, and it is tempting to speculate that exercise training beneficially affects these variables too [79]. Importantly, the field of exercise physiology has been boosted in recent years by the identification of the exercising muscle as an important endocrine organ that secretes so-called myokines, which are involved in inter-organ communication. Recent discoveries include the hormones irisin (although controversial), meteorin-like, Angptl4 and β-aminoisobutyric acid [80–82]. In accordance, these myokines have been suggested to affect whole-body energy metabolism, including activation of brown or beige adipocytes, reduce inflammation and improve hepatic fat oxidation. For example, Angptl4 was identified as a novel myokine that regulates excessive fat storage in non-exercising muscle and the heart [83], suggesting that increasing energy turnover in the active muscle may also affect substrate turnover in non-exercising muscles. Clearly, more studies are needed in humans to unravel how exercise-induced increases in energy turnover may affect metabolism in tissues and organs other than skeletal muscle. This may open an entirely new outlook on understanding how exercise may be able to improve health in general.

### Exercise mimetics

The beneficial effects of exercise on metabolic health have stimulated the search for drugs or nutritional compounds that can mimic the effects of exercise. Although no pill could be expected to induce all health effects of exercise, opportunities lie in the molecular pathways that are central to much of the exercise-induced improvements in metabolic health. Resveratrol is one such nutritional compound, found in red wine, among other things, which has been extensively studied in pre-clinical experiments and has also been tested in humans. Resveratrol can activate the SIRT1–AMPK–PGC1 axis, and could thereby be described as an exercise-mimetic (Fig. 2). Although results are not wholly consistent, clinical trials in type 2 diabetes patients imply that resveratrol has a glucose-lowering effect (for review see [84]). Unfortunately, the number of human interventions with molecular details is so far limited. We have previously shown that resveratrol can indeed activate AMPK and increase SIRT1 and PGC1 levels in human skeletal muscle and results in elevated mitochondrial function and reduced hepatic fat content [40]. Interestingly and consistent with the outline above, resveratrol intake also resulted in increased intramyocellular lipid content and PLIN expression [85], suggesting that not only oxidative capacity but also the capacity for energy turnover is boosted and linked to improved metabolic health, as is the case with exercise training. However, resveratrol also reduces the RMR and the long-term consequences of this would need to be established. So far, the number of clinical trials using resveratrol or other AMPK/SIRT1 targets is still very small but this is expected to change rapidly in the coming years.

### Cold exposure

Environmental temperature can have a major effect on whole-body and cellular energy expenditure (Fig. 1). When body heat loss is substantially increased by exposure to the cold, high rates of lipid and carbohydrate oxidation are essential to maintain an increased metabolic rate. Indeed, severe cold exposure in animals has been shown to increase lipolysis, lipid oxidation and NEFA turnover (among others: [86]), as well as glucose oxidation and turnover, and thereby improves glucose tolerance and peripheral glucose uptake (Fig. 2) [87, 88]. These findings indicate that an increase in cold-induced shivering thermogenesis can have pronounced effects on glucose homeostasis; however, these studies were conducted under severe cold exposure, a condition that cannot be sustained for long periods of time.

#### Role of NST

In contrast to extreme cold, mild cold exposure is an intervention that is feasible for application in humans for longer periods of time. When humans and animals are exposed to milder cold, NST increases to produce heat. Interestingly, this NST is blunted in obese individuals and is significantly reduced when compared with NST in lean counterparts [89]. This relatively low NST may be related to body composition, as overweight and obese individuals have much more (subcutaneous) body fat and have more tissue insulation. Therefore, in daily (indoor) situations obese people experience much less cold than their lean counterparts, thereby triggering NST to a lesser extent. Indeed, after weight loss, morbidly obese patients show an increased NST capacity [90]. In older people, NST is reduced and, together with reduced body temperatures in the cold, their net energy expenditure in the cold may even be lower than at thermoneutral conditions [17]. The extent to which reduced NST in the elderly is caused by habituation or biological factors related to ageing is not known. Older people in western society generally tend to spend more time indoors in relatively warm and stable environments and are less tolerant of lower ambient temperatures. Therefore they may have lost their NST capacity. Both by living in such a protective stable environment and by increased body fat, NST and related metabolic processes are diminished. On the other hand, biological ageing itself may also affect their metabolic cold responses. Intriguingly, NST is also blunted in patients with type 2 diabetes [91]. However, the lower NST in type 2 diabetes may be related to the fact that most type 2 diabetes patients are older and overweight. It is currently not known whether a low NST plays a role in the aetiology of type 2 diabetes.

#### Role of brown adipose tissue

In rodents the main tissue responsible for NST is brown adipose tissue (BAT) [92]. Although NST in humans had been reported earlier, its relationship to functional BAT in adult humans was not revealed until 2009 [93–96]. In contrast to white adipose tissue, BAT burns triacylglycerol and glucose to generate heat through mitochondrial uncoupling [92]. Cold is the main stimulator of sympathetic nervous system-mediated BAT activation. Human brown fat is mainly studied by fluorodeoxyglucose–PET/CT imaging, which shows glucose uptake (rate) in those tissues that use glucose [97]. BAT is not activated in fasting and thermoneutral conditions [98], while it is activated by mild cold (i.e. without shivering) [94]. Using the appropriate individual cooling protocols [99], BAT glucose uptake was found to be significantly related to NST, indicating a role for BAT in whole-body thermogenesis, as shown in rodent studies. In parallel with NST, cold-induced BAT activation is reduced in obese and elderly individuals and those with type 2 diabetes [91, 100, 101]. Only recently, studies on the effect of cold acclimation on NST and BAT activity have been performed. Cold acclimation by intermittent exposure to a cool (14–17°C), or cold (10°C) environment resulted in significant increases in NST capacity [102]. A 10 day cold acclimation study with 6 h exposure to 14–15°C per day was enough to significantly increase NST by 65% on average [103]. A 6 week mild cold acclimation study (daily 2 h cold exposure at 17°C) also resulted in an increase in NST together with a concomitant decrease in body fat mass [104]. The latter two studies also revealed significant increases in BAT presence and activation [103, 104]. All in all, cold-induced BAT activity is significant in adults and parallels NST. The actual quantitative contributions of BAT and of other tissues (e.g. skeletal muscle) to whole-body NST are, however, not elucidated and await further studies. Furthermore, more information is needed on the duration, timing and temperatures to find out which treatments are most effective with respect to increasing NST.

Whether activation of BAT (potentially via elevating NST) affects glucose homeostasis and insulin sensitivity has not been studied extensively. Thus, glucose is oxidised in high amounts by BAT when activated, although the direct contribution of glucose oxidation to total thermogenesis in BAT is believed to be relatively small compared with that of fat oxidation, somewhere in the range of 10–16% [92, 105, 106]. It is likely that the glucose that is taken up is mainly used for the synthesis of glycerol-3-phosphate and triacylglycerols and also for the supply of extramitochondrial ATP through glycolysis to support fatty acid esterification to triacylglycerol and other cellular functions [107]. A study on noradrenaline (norepinephrine) stimulation of rat brown adipocytes revealed that glucose uptake and oxygen consumption were related. It has also been shown that increasing BAT by transplantation in mice has advantageous effects on body composition, insulin sensitivity and glucose metabolism [108].

In humans, retrospective patient studies show inverse relationships between BAT activity and diabetes and glycaemia [96, 109]. Prospective cold exposure studies show, as mentioned above, that glucose uptake in BAT is positively related to NST [103, 110]. These observations show that cold-induced thermogenesis goes hand in hand with increased glucose metabolism. The acute cold glucose uptake rate per unit of tissue mass as determined by dynamic PET/CT was higher in BAT than in skeletal muscle [110, 111]. Interestingly, insulin-stimulated glucose uptake in BAT in humans was positively related to the *M* value (a measure of whole-body insulin sensitivity derived from hyperinsulinaemic–euglycaemic clamps) [112].

Whether the increased uptake of glucose by BAT significantly contributes to whole-body glucose metabolism in type 2 diabetes has not yet been substantiated, although it was recently shown that individuals with active BAT, when compared with individuals without BAT, showed significantly increased resting energy expenditure, whole-body glucose disposal, plasma glucose oxidation and insulin sensitivity [113]. In another study, Lee et al [114] showed that staying overnight in cold chambers (19°C) for 1 month increased BAT activity together with improved postprandial insulin sensitivity. In a recent study in individuals with type 2 diabetes we studied the effect of cold acclimation on BAT activity and insulin sensitivity using hyperinsulinaemic–euglycaemic clamp. The cold acclimation protocol was identical to that used by van der Lans et al [103], where we found significant increases in BAT and NST. In type 2 diabetes patients the amount of BAT at baseline was significantly lower than that in healthy lean individuals [91]. Acclimation increased BAT activity significantly but levels were still very low [91]. Very interestingly, insulin sensitivity increased after cold acclimation by 43% on average [91]. It is very unlikely that the small increase in BAT activity could be responsible for this improved insulin sensitivity. In fact, the study showed that the improved insulin sensitivity could be explained by enhanced GLUT4 translocation in skeletal muscle in the basal state, an effect that had been previously observed in cold-acclimated rats [115] and has been confirmed in obese humans [116]. Although the mechanisms responsible for GLUT4 translocation upon cold stimulation remain to be elucidated, these findings clearly demonstrate that the significant improvement in insulin sensitivity can be attributed to skeletal muscle tissue, rather than to BAT, and may involve increased energy turnover. However, since BAT increased in all participants after cold acclimation, an indirect role for BAT (e.g. by secreting BATokines) cannot be fully excluded.

## Conclusions

In conclusion, energy metabolism in humans can be affected by exercise and cold-exposure interventions. Such interventions are associated with metabolic health effects that cannot be explained by effects on body-weight regulation. Rather, increasing energy turnover activates cellular energy sensors, such as AMPK, which trigger beneficial adaptive responses. Although the exact underlying mechanisms are still not fully understood in humans, exercise and mild cold exposure provide strong intervention strategies for the prevention and treatment of type 2 diabetes mellitus.

## Electronic supplementary material

Below is the link to the electronic supplementary material.

Electronic supplementary material (Downloadable slideset (ESM) (PDF 452 kb)

## Abbreviations

ANT1: Adenine nucleotide translocase 1
BAT: Brown adipose tissue
BMR: Basal metabolic rate
DIT: Diet-induced thermogenesis
DNP: 2,4-Dinitrophenol
NEN: Niclosamide ethanolamine salt
NST: Non-shivering thermogenesis
PLIN: Perilipin
PGC1: Peroxisome proliferator-activated receptor γ coactivator 1
RMR: Resting metabolic rate
SIRT1: Sirtuin 1
