# Supplementary material for: Combatting type 2 diabetes by turning up the heat
Source: Diabetologia. 2016 Sep 3;59(11):2269–79. doi: 10.1007/s00125-016-4068-3 (PMC5506100; doi:10.1007/s00125-016-4068-3)
Supplement: Supplementary file 1 — (Downloadable slideset (ESM) (PDF 452 kb) [file 125_2016_4068_MOESM1_ESM.pdf]

# Interventions targeting energy turnover can affect metabolic health

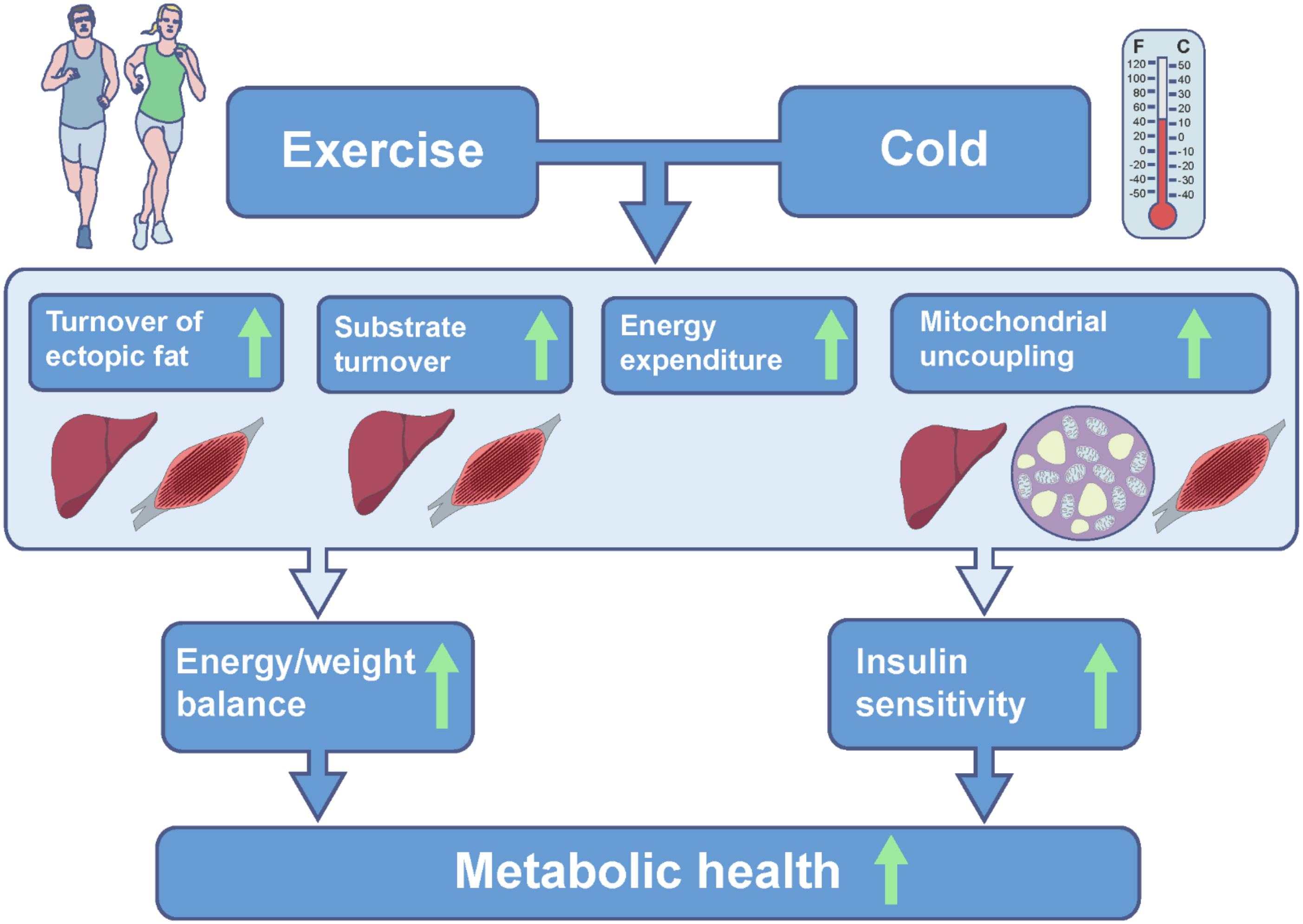


© Schrauwen and van Marken Lichtenbelt 2016
Diabetologia DOI 10.1007/s00125-016-4068-3

Diabetologia

# Cellular mechanisms linking energy-boosting interventions to metabolic health effects

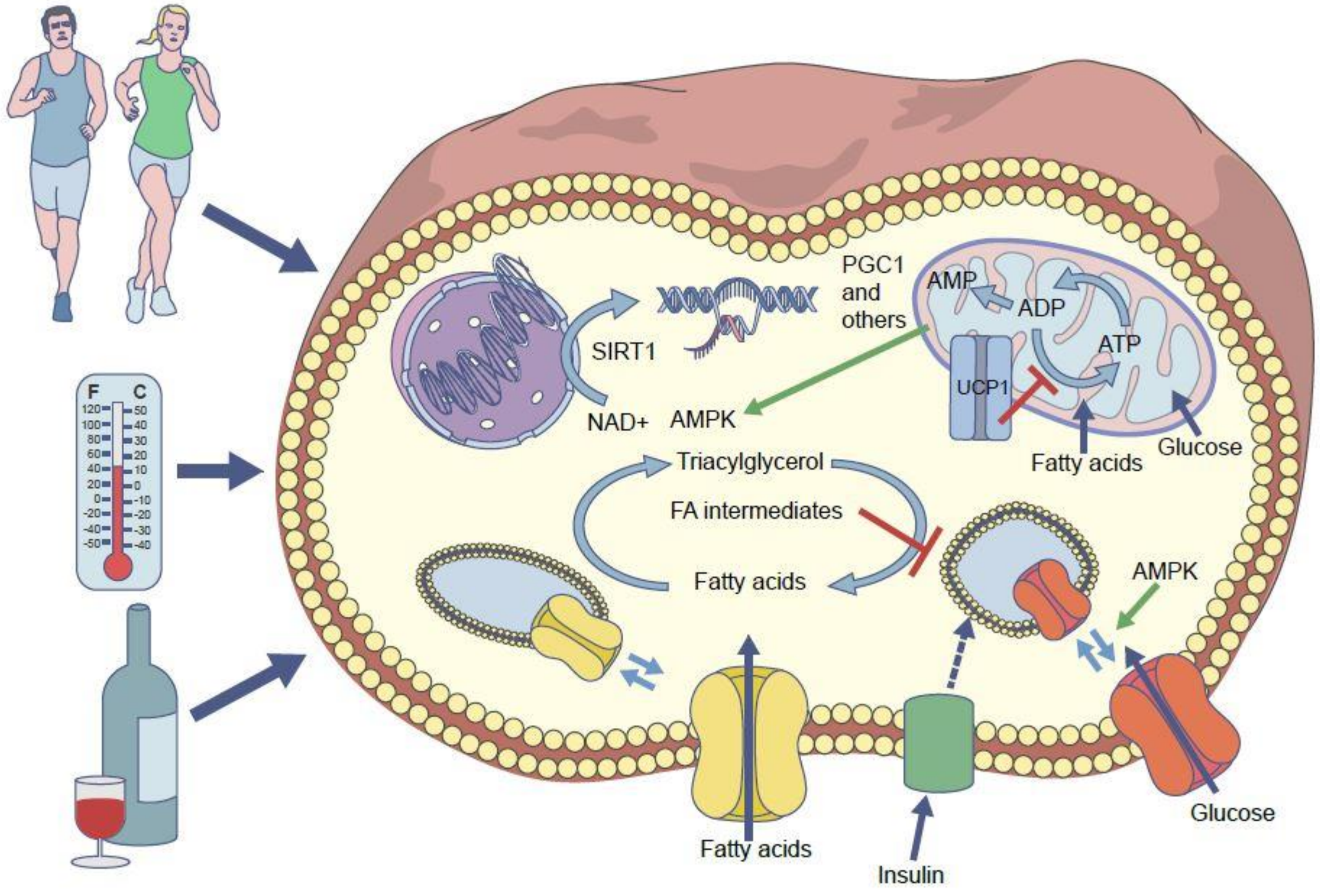


© Schrauwen and van Marken Lichtenbelt 2016
Diabetologia DOI 10.1007/s00125-016-4068-3

Diabetologia
